# Risk factors for inguinal lymph node metastasis in patients with penile cancer

**DOI:** 10.1590/S1677-5538.IBJU.2021.0613.1

**Published:** 2022-03-01

**Authors:** Leandro Koifman

**Affiliations:** 1 Serviço de Urologia Hospital Municipal Souza Aguiar Rio de Janeiro RJ Brasil Serviço de Urologia, Hospital Municipal Souza Aguiar, Rio de Janeiro, RJ, Brasil

## COMMENT

Although penile cancer is a global health problem, especially in underdeveloped countries, with high annual expenses for care of patients affected by the disease, few healthcare policy efforts have been taken by authorities to mitigate this devastating disease. The psychological and physical burden that arises from the treatment, as well as the demoralizing demise that comes with advanced illness, has been widely neglected by the urology scientific community in favor of other urological malignancies. In this scenario, rare investigators, without adequate research funds and governmental support, have been responsible for the few advances in the field of investigation and treatment of penile cancer in recent decades.

In the current edition of the International Brazilian Journal of Urology, the authors analyze thorough a cross-sectional study, the risk factors for inguinal lymph node metastasis in patients with penile cancer (1). It is well established that the presence and extent of inguinal metastases are the most important prognostic factors related to survival of patients with penile carcinoma (2). Although this carcinoma is potentially curable by radical inguinal lymphadenectomy, the procedure is historically associated with a significant incidence of morbidity, leaving many patients at risk for inguinal metastases without the appropriate approach (3). The question raised by urologists over the last 40 years remains the same: Should inguinal lymphadenectomy be performed in all patients, exposing them to the risks of inguinal dissection? Unfortunately, the question still remains without a definitive answer. At initial presentation, about 50% of patients with penile cancer have clinically detectable inguinal lymphadenopathy. However, only half of them have metastatic lymph node involvement. On the other hand, about 20% of patients with clinically negative inguinal lymph nodes have micrometastasis that will only be diagnosed through histopathological examination of surgical specimens obtained from lymphadenectomy. Thus, inguinal lymphadenectomy is unnecessary in 80% of patients with clinically negative lymph nodes and 50% of those with clinically positive lymph nodes (4–6).

In order to determine the best candidates to perform inguinal lymphadenectomy, the authors present in the current study an attempt to stratify patients at high risk for developing inguinal metastases by analyzing independent risk factors. The results demonstrate that patients with low to moderate tumor differentiation (p=0.009), lymphovascular invasion (p=0.025) and T-stage 2 (p=0.010) or higher are more likely to develop inguinal lymph node metastasis. These results are not surprising. Indeed, they are in line with data from previous published studies and the current guidelines on penile carcinoma, advocating the performance of prophylactic radical inguinal lymphadenectomy for patients considered at intermediate and high risk of inguinal lymphatic spread (4–8). An interesting finding of the study concerns the maximum diameter of enlarged inguinal nodes (>1.5 cm) measured by imaging, which was found to be an independent risk factor (p= 0.045) for inguinal metastasis. Palpable lymph nodes are strongly indicative of metastasis and should be promptly treated with radical inguinal lymphadenectomy (2, 4, 5). Furthermore, it is reasonable to assume that lymph nodes with diameter >1.5 cm can be identified by simple physical examination, leaving imaging methods in such cases to staging or evaluation of obese patients in cases where physical examination is unreliable (5). Despite the need of further investigation to support this finding, it can be helpful in decisions to perform inguinal dissection, especially in doubtful cases, such as in obese patients with pT1G1 primary tumor pathology.

Despite the advances in the treatment of penile carcinoma, efforts to fight the development of this malignancy are still far from desirable. The engagement of the international urological community in association with local authorities, adequate funding for research and improvement of the human development index, especially in underdeveloped countries, seem to be the best ways to mitigate the disease.
